# Supplementary material for: Unveiling Angiotensin II and Losartan-Induced Gene Regulatory Networks Using Human Urine-Derived Podocytes
Source: Int J Mol Sci. 2023 Jun 23;24(13):10551. doi: 10.3390/ijms241310551 (PMC10341883; doi:10.3390/ijms241310551)
Supplement: Supplementary file 1 [file ijms-24-10551-s001.zip › Supplementary Materials_R1.pdf]

Supplemental Material Table of Contents

Supplementary Table S1: Used antibodies.

Supplementary Table S2: Used qPCR primers.

Supplementary Table S3: gostats

Supplementary Table S4: full gene list

Supplementary Table S5: KEGG signaling pathways.

Supplementary Figure S1: uncropped western blot images.

Supplementary Figure S2: Hierachical and heatmap.

Supplementary Figure S3: KEGG signaling pathways of Oxytocin signaling pathway, renin secretion and vascular smooth muscle contraction.

Supplementary Table S1: Used antibodies

**Primary Antibody**	**Specificity**	**Dilution**<br>**WB**	**Manufacturer**
**GAPDH**	Rabbit	1:4000	Thermo Fisher Scientific Dreieich, Germany
**AGTR1**	Rabbit	1:1000	Novusbio, Wiesbaden, Germany
**AGTR2**	Rabbit	1:1000	Novusbio, Wiesbaden, Germany
**NPHS1**	Rabbit	1:200 (IF)<br>1:1000	Thermo Fisher Scientific, Waltham, USA (PA5-20330)
**Secondary Antibody**	Specificity	Dilution	Manufacturer
**Goat anti-Rabbit IgG secondary Antibody HRP**	Rabbit	1:1000	Thermo Fisher Scientific, Dreieich, Germany
**Alexa 555**	Rabbit	1:500	Thermo Fisher Sci, Dreieich, Germany

Supplementary Table S2: Used qPCR primers

**Primer**	**Sequence** (5' → 3')	**Predicted size [bp]**
**RPL0**	F: TCGACAATGGCAGCATCTAC<br>R: ATCCGTCTCCACAGACAAGG	195
**AGTR1**	F: TCTCAGCATTGATCGATACC<br>R: TGACTTTGGCTACAAGCATT	60
**AGTR2**	F: TATGGCCTGTTTGTCCTCAT<br>R: CATTGGGCATATTTCGG	80
**NPHS1**	F: GACCCCTCTACGATGAAGTGC<br>R: GGGTTCCAGAGTGTCCAAGTC	119

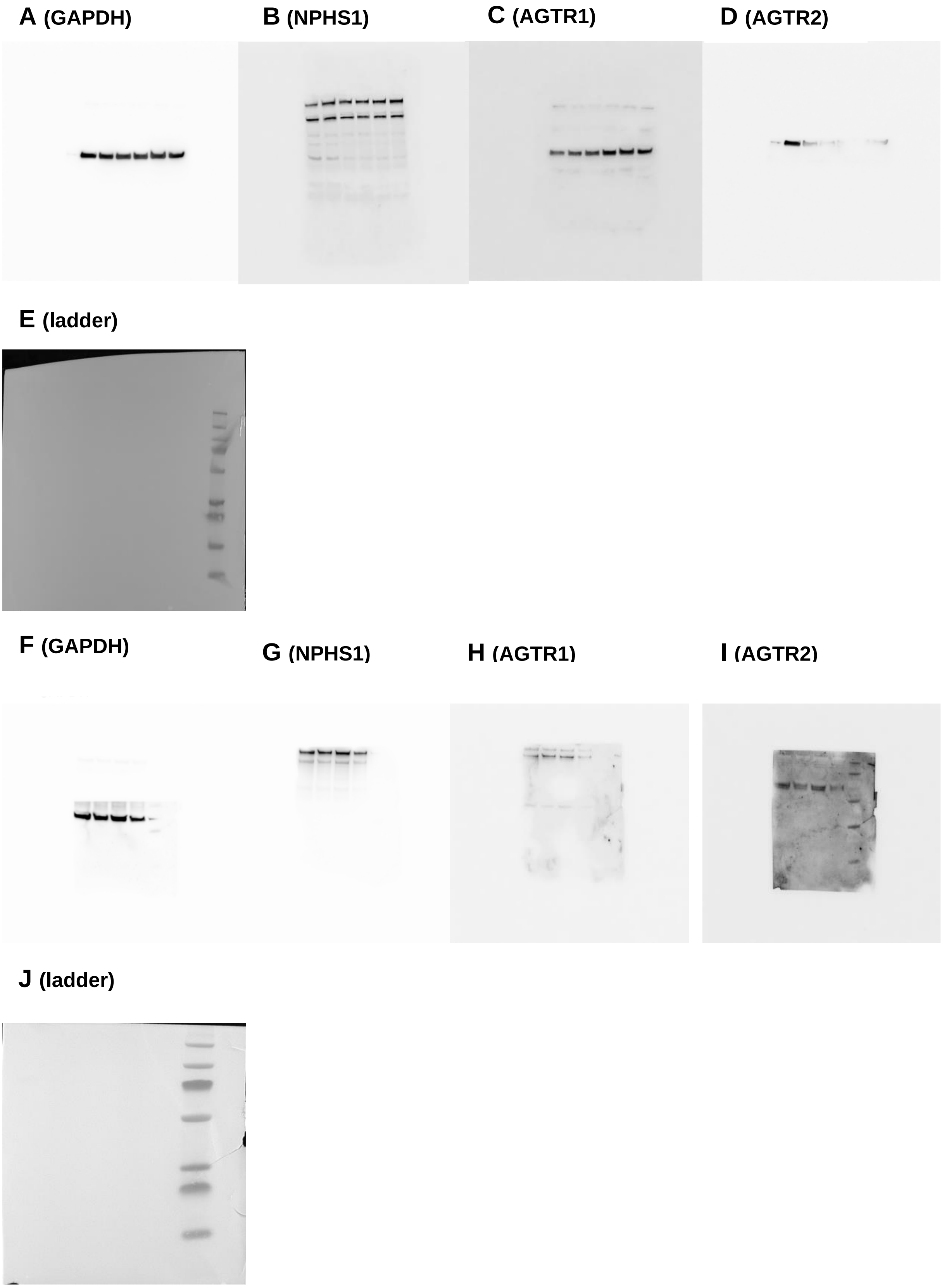

Supplementary Figure S1: Uncropped western blot images

The detected proteins are presented from left to right: A+F = GAPDH, B+G = NPHS1, C+H = AGTR1, D+I = AGTR2. Supplementary figure E+J represents the ladder for the western blot images.
The loading scheme starting next to the ladder from the right for the western blot represented in supplementary figure 1A-D is as followed:
UM51hTERT control, UM51hTERT treated with100 µM Angiotensin II (ANG II) for 24h, UM51hTERT treated with 1 µM + 0.01 µM losartan and UM51hTERT treated with the combination of 1 µM + 0.01 µM 24 h losartan treatment followed by a 100 µM 24 h ANG II treatment.
The loading scheme starting next to the ladder from the right for supplementary figure F-I is as followed: UM51 podocyte control. This is followed by UM51 podocyte treated with 100 µM Angiotensin II (ANG II) for 24h, UM51 podocyte treated with 1µM losartan and UM51 podocyte treated with the combination of 1 µM losartan for 24h followed by a 100 µM 24 h ANG II treatment.

A

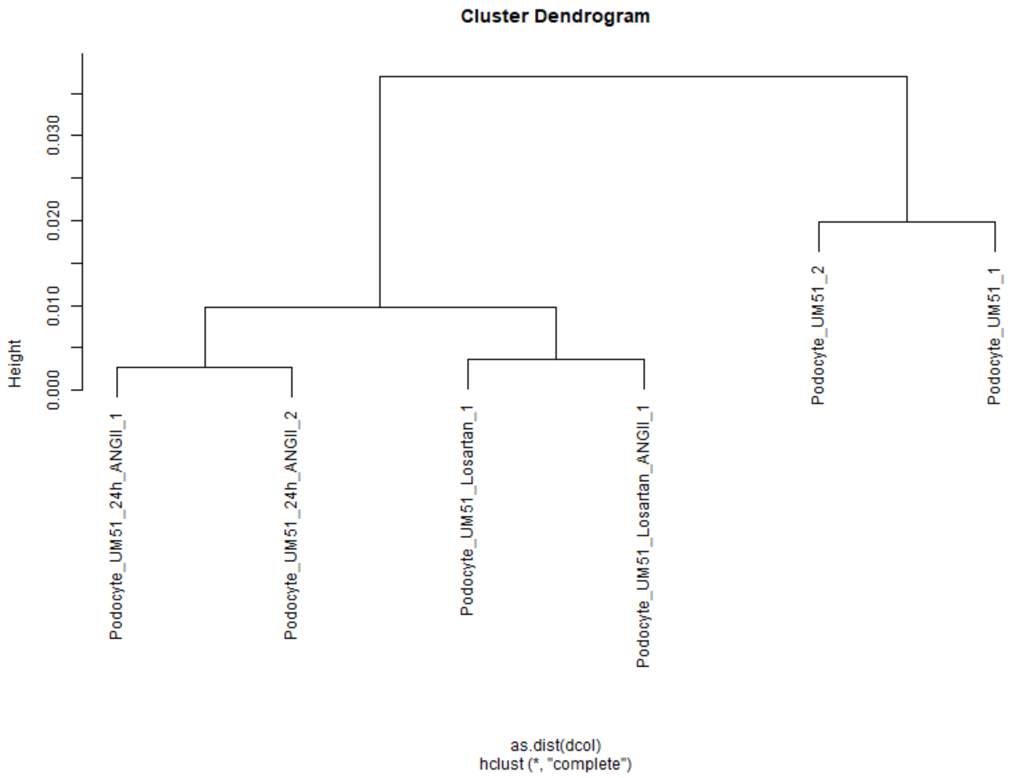

B

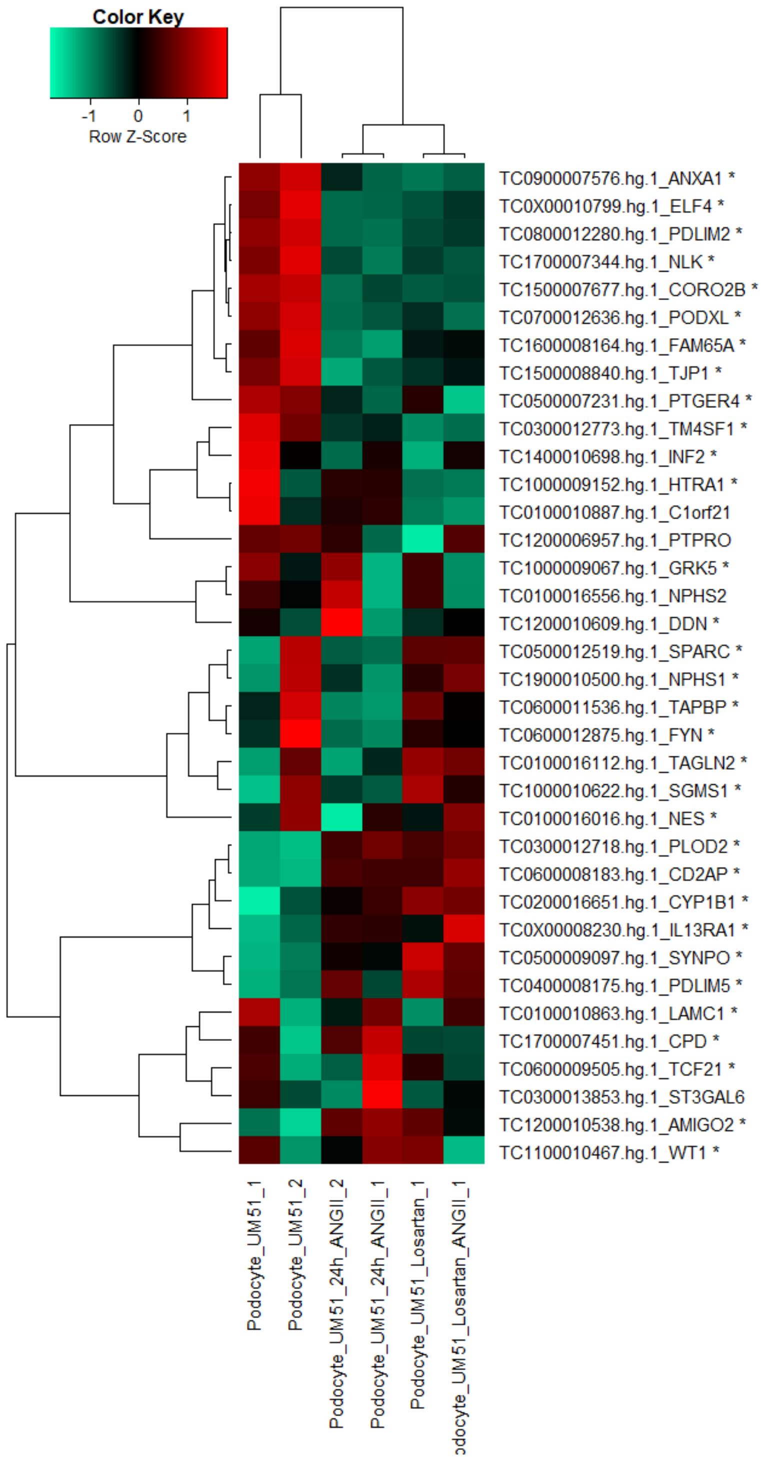

Supplementary Figure S2: Hierachical cluster dendogram and heatmap.
The hierarchical cluster dendogram and the heatmap are based on transcriptomes of untreated human urine derived podocytes and stimulated with 100 µM ANG II or 1 µM Losartan or the combination of 100 µM ANG II and 1 µM Losartan. The heatmap (B) includes all podocyte specific genes which were expressed in the several treatments.

A

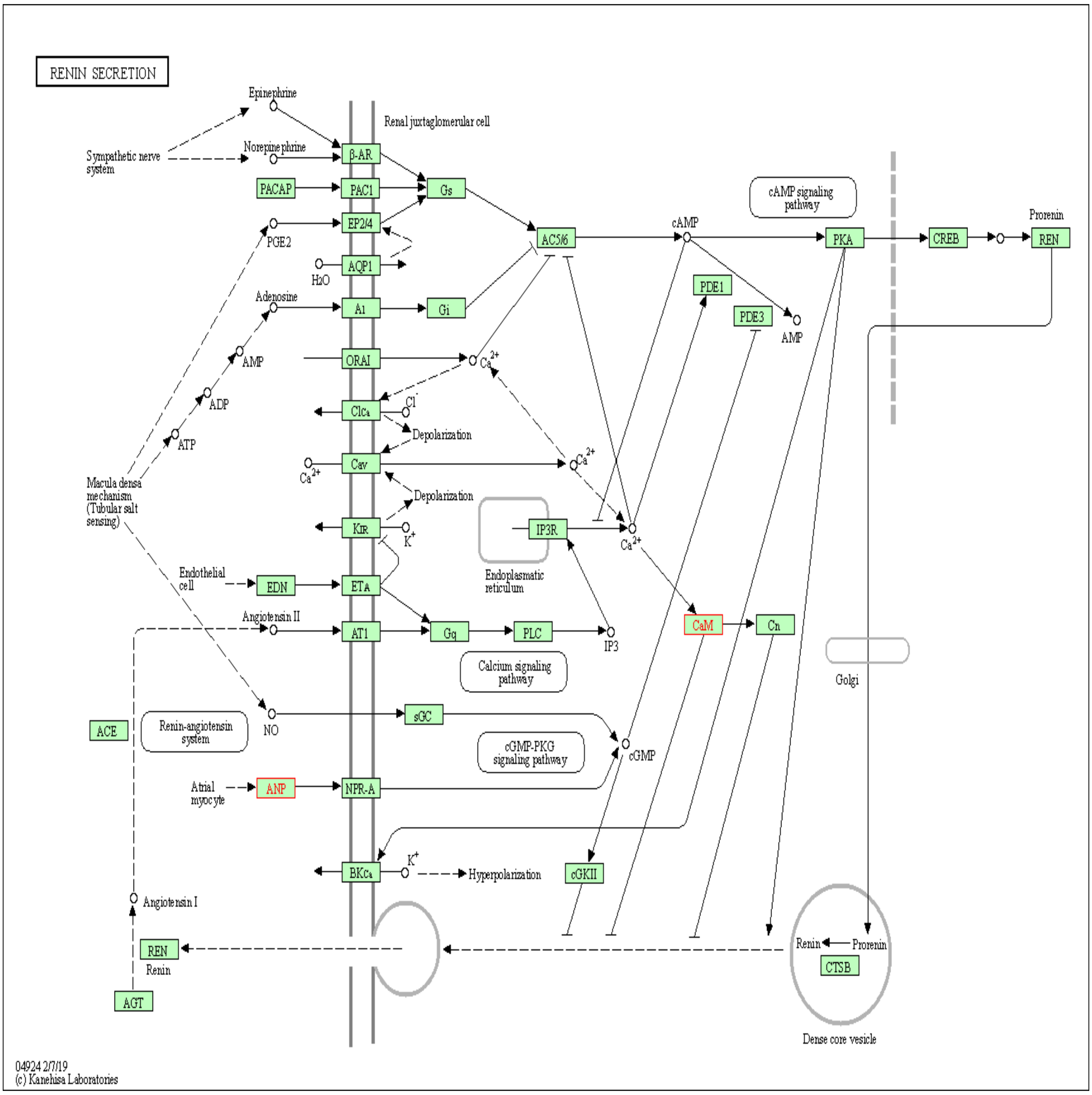

Supplementary Figure S3 continuing.

B

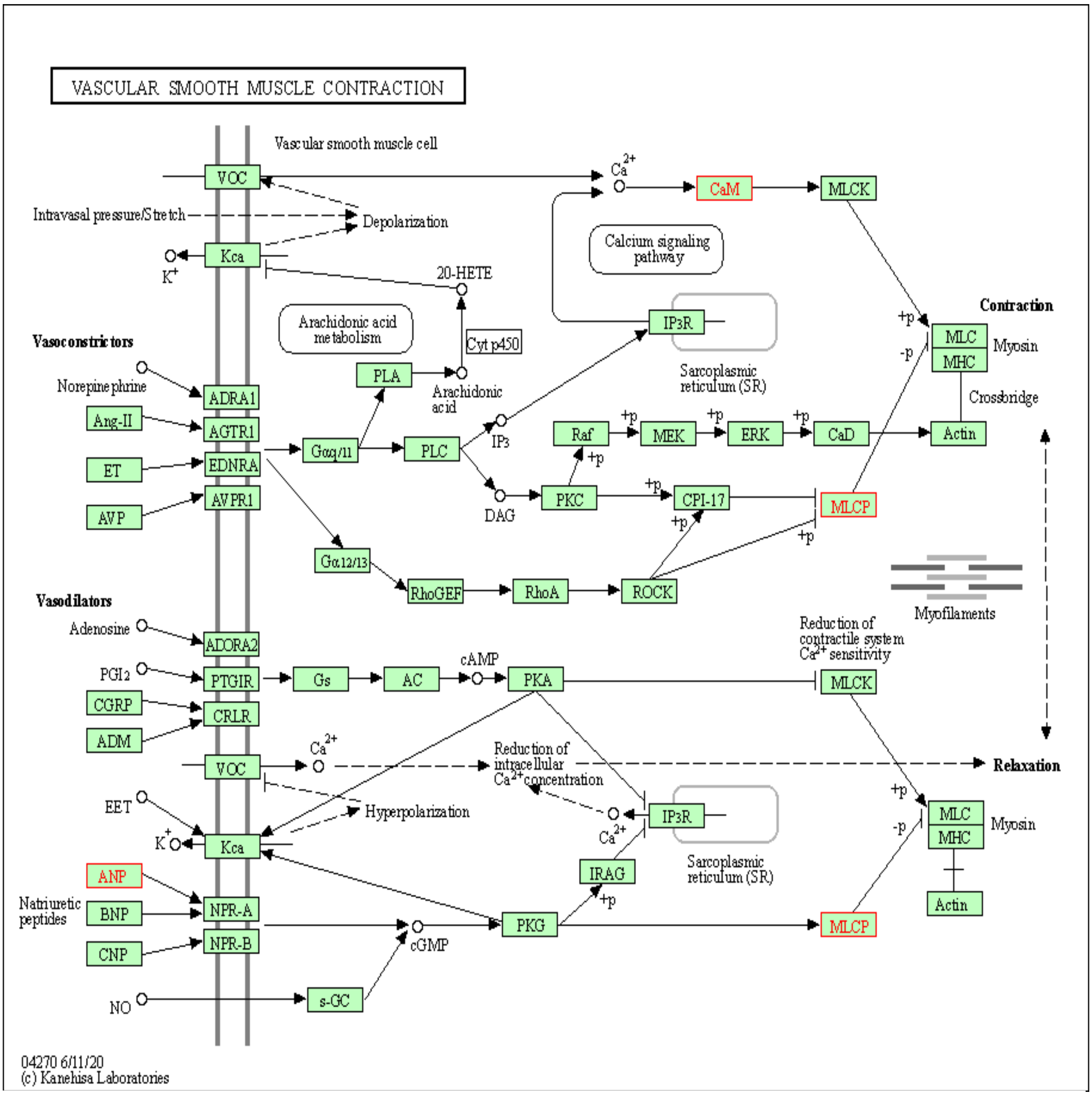

Supplementary Figure S3 continuing.

A

C

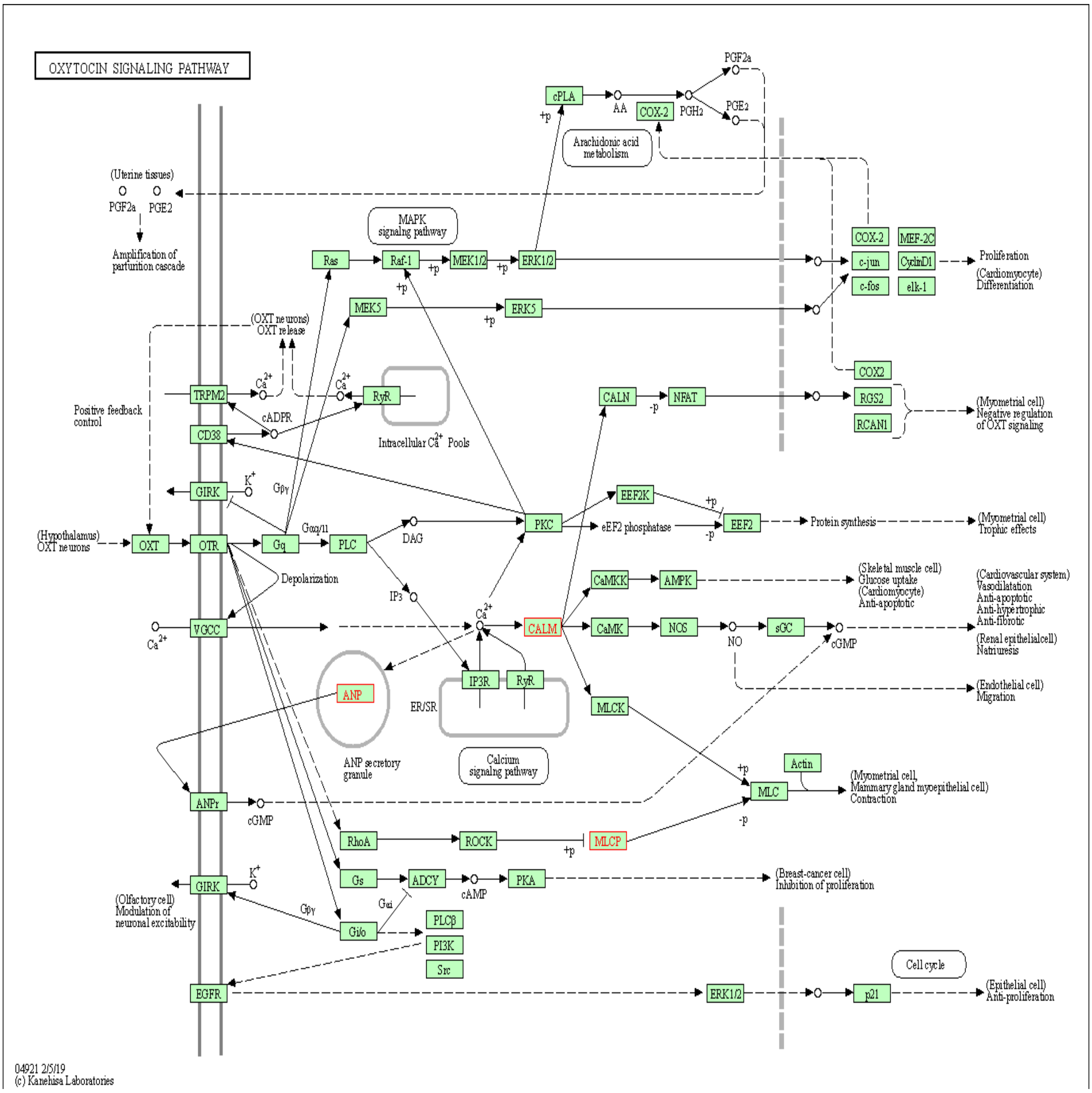

Supplementary Figure S3: KEGG signaling pathways of Oxytocin signaling pathway, renin secretion and vascular smooth muscle contraction.
